# Diversity of the Bacterial Microbiome in the Roots of Four *Saccharum* Species: *S. spontaneum*, *S. robustum*, *S. barberi*, and *S. officinarum*

**DOI:** 10.3389/fmicb.2018.00267

**Published:** 2018-02-21

**Authors:** Meng Dong, Zongtao Yang, Guangyuan Cheng, Lei Peng, Qian Xu, Jingsheng Xu

**Affiliations:** ^1^Key Laboratory of Sugarcane Biology and Genetic Breeding, Ministry of Agriculture, Fujian Agriculture and Forestry University, Fuzhou, China; ^2^Key Laboratory of Ministry of Education for Genetics, Breeding and Multiple Utilization of Crops, College of Crop Science, Fujian Agriculture and Forestry University, Fuzhou, China

**Keywords:** 16S rRNA, gene sequencing, *nifH*, diazotroph, endophytic bacteria, sugarcane

## Abstract

Endophytic bacteria are nearly ubiquitously present in the internal tissues of plants, and some endophytes can promote plant growth. In this study, we sampled the roots of four ancestral species of sugarcane (two genotypes per species) and two sugarcane cultivars, and used 16S rRNA and *nifH* gene sequencing to characterize the root endophytic bacterial communities and diazotroph diversity. A total of 7,198 operational taxonomic units (OTUs) were detected for the endophytic bacteria community. The endophytic bacterial communities exhibited significantly different α- and β-diversities. From the 202 detected families in the sugarcane roots, a core microbiome containing 13 families was identified. The *nifH* gene was successfully detected in 9 of 30 samples from the four sugarcane species assayed, and 1,734 OTUs were merged for endophytic diazotrophs. In the tested samples, 43 families of endophytic diazotrophs were detected, and six families showed differences across samples. Among the 20 most abundant detected genera, 10 have been reported to be involved in nitrogen fixation in sugarcane. These findings demonstrate the diversity of the microbial communities in different sugarcane germplasms and shed light on the mechanism of biological nitrogen fixation in sugarcane.

## Introduction

Most crop plants grow in close association with microbial communities, which can be divided into three groups, endophytic, epiphytic, or closely associated. Plants that grow in soil live in close proximity to an abundance of diverse microbes (Tringe et al., 2005), and symbioses between plants and associated microbes serve to benefit both partners. The plants benefit from these relationships as key nutrients are altered into more usable forms by the microbes before being assimilated by plants (Long, 1989; Bolan, 1991; Zhang et al., 2009). In turn, the plants provide carbon metabolites as root exudates to the endophytes and bacteria in the rhizosphere (Bais et al., 2006; Lopes et al., 2016). Plant-associated microbes can also protect plants against phytopathogens (Innerebner et al., 2011), improve plant growth through the production of phytohormones (Ali et al., 2009), and help plants withstand heat (Castiglioni et al., 2008), salt (Zhang et al., 2008), and other stresses.

Sugarcane (*Saccharum* spp. hybrid) is used as a major source for sugar and biofuel production worldwide, and it is one of the most economically significant crops (Liu et al., 2010; Heller-Uszynska et al., 2011). To increase sugarcane yield, high rates of synthetic nitrogen fertilizer are typically applied, especially in India and China (Wood et al., 2010; Robinson et al., 2011). However, high-dose applications of synthetic nitrogen fertilizer cause serious environmental pollution (Li and Yang, 2015). Therefore, manipulation of the nitrogen fixation capacity of the sugarcane-associated microbiome has emerged as an alternative to nitrogen fertilizer application and has been extensively studied, especially for endophytic diazotrophs (Elbeltagy et al., 2001; Reis et al., 2004; Lin et al., 2014; De Souza et al., 2016; Proença et al., 2017). Many endophytic diazotrophs have been isolated and identified by culture-dependent methods, such as *Gluconacetobacter diazotrophicus*, *Herbaspirillum rubrisubalbicans*, *Herbaspirillum seropedicae*, *Klebsiella* spp. (Magalhaes et al., 1983; Cavalcante and Dobereiner, 1988; Baldani et al., 1996; James et al., 1997; Elbeltagy et al., 2001), *Pseudomonas koreensis* and *Pseudomonas entomophila* (Li et al., 2017), *Nitrospirillum amazonense*, and *Paraburkholderia tropica* (Kaur et al., 2016). Cloning and sequencing of the 16S rRNA genes of the endophytic communities in the stems of sugarcane cultivars revealed sequences similar to those from several genera, including *Pseudomonas*, *Enterobacter*, *Pantoea*, *Serratia*, *Citrobacter*, and *Klebsiella* (Magnani et al., 2013). With the development of next-generation sequencing technologies, culture-independent methods have been employed to determine the profiles of the sugarcane-associated microbial communities by 16S rRNA sequencing (De Souza et al., 2016; Yeoh et al., 2016). In addition, there has been a focus on sequencing and identifying the *nifH* gene to isolate and characterize the diazotrophic community (Rouws et al., 2014; Li et al., 2017).

Recent studies showed that application of nitrogen fertilizer did not change the core microbial community in sugarcane cultivars, and sugarcane variety (plant genotype) had only a subtle effect on community composition (Yeoh et al., 2016). In addition, the richness, but not the abundance, of the endophytic bacterial community did not vary among the different organs of sugarcane (De Souza et al., 2016). However, such previous studies were mainly conducted on commercial sugarcane cultivars. Studies using the model system *Arabidopsis thaliana*, cultivated under controlled conditions in natural soils, indicated that host genotype has a small but measurable effect on the microbes inhabiting the endophyte compartment of the root (Bulgarelli et al., 2012; Lundberg et al., 2012). However, the profiles of endophytic bacteria from different sugarcane species have not been characterized.

In this study, eight ancestral varieties and two commercial cultivars of sugarcane were used to investigate the culture-independent profiles of endophytic bacteria during the period of rapid growth by 16S rRNA and *nifH* gene amplification and sequencing. This study allowed us to evaluate the effects of sugarcane genotype on the diversity of endophytic bacteria and diazotrophs.

## Materials and Methods

### Sugarcane Germplasm and Microbiome Sample Collection

Four *Saccharum* species, *S. officinarum* (Black Cheribon and Badila), *S. robustum* (51NG208 and FJDY), *S. barberi* (HATUNI and Katha), *S. spontaneum* (HN-83 and FJ88-1), and two commercial sugarcane cultivars (ROC22 and YT93-159) were planted in the National Nursery of Sugarcane Germplasm Resources (NNSGR, Kaiyuan, China). Conventional culturing practices were used as follows: N, 350 kg/ha; P_2_O_5_, 105 kg/ha; and K_2_O, 115 kg/ha. The composition of the experimental soil was as follows: 20.8 g/kg organic matter, 1.58 g/kg total nitrogen, 0.61 g/kg total phosphorus, 13.3 g/kg total potassium, 78.87 mg/kg available nitrogen, 10.31 mg/kg available phosphorus, 99.86 mg/kg available potassium, and pH 6.2.

The roots were sampled in July when the sugarcane plants were in the elongation stage. For each species or cultivar, three plants were selected, and one root sample was collected from each plant. Roots with white tips, which is indicative of active growth, were partially excavated in the row shoulder from the upper 15–20 cm of soil. Root samples were standardized by taking the first 10 cm from the root tip. The samples were washed with water and sterilized, first with 75% alcohol and then with a sodium hypochlorite solution containing 1% active chlorine. Next, the samples were washed with sterilized water, and cleaned using sterilized filter paper, and placed into 50-mL sterilized centrifuge tubes. Then, the samples were frozen at -80°C and stored until use.

### DNA Extraction and Construction of Sequencing Libraries

Total bacterial genomic DNA was extracted from specimens using the E.Z.N.A.^®^ Stool DNA kit (Omega Bio-tek, Norcross, GA, United States). Then a DNA library was constructed from the extracted genomic DNA and sequenced by REALBIO TECHNOLOGY (Shanghai, China). Microbial 16S rRNA was amplified with the index and adaptor-linked universal primers 341F (ACTCCTACGGGAGGCAGCAG) and 806R (GGACTACHVGGGTWTCTAAT), which target the V3–V4 region. The *nifH* gene was amplified with primers Pol-F (TGCGAYCCSAARGCBGACTC) and Pol-R (ATSGCCATCATYTCRCCGGA) as previously reported (Poly et al., 2001).

PCR was performed using diluted genomic DNA as template with the KAPA HiFi Hotstart PCR kit (Sigma-Aldrich, Boston, MA, United States) and a high fidelity enzyme to ensure the accuracy and efficiency of amplification. The PCR products were detected by using 2% agarose gel electrophoresis and were recovered with the AxyPrep DNA gel Recovery kit (AXYGEN). Amplicon libraries were quantified using a Qubit 2.0 Fluorometer (Thermo Fisher Scientific, Waltham, MA, United States) to pool at equal concentrations and were sequenced on an Illumina HiSeq 2500 platform (Illumina, San Diego, CA, United States) to obtain paired-end reads of approximately 250 bp.

### Microbial Community Profile Data Processing and Statistical Analysis

The paired-end reads were merged into longer contigs and quality filtered to remove tags with lengths <220 nt, average quality scores of <20, and tags containing >3 nitrogenous bases by PANDAseq. After discarding singletons, the high-quality tags were clustered into operational taxonomic units (OTUs) by Usearch, using a similarity threshold of 0.97. The OTUs were further subjected to a taxonomy-based analysis using an RDP algorithm and the RDPII database. A heat map was created using R, and cluster analysis was performed with Usearch. The α- (Simpson, Shannon, and Chao-1) and β-diversities were analyzed using QIIME (Caporaso et al., 2010; Kuczynski et al., 2011). Linear discriminant analysis (LDA) and effect size (LEfSe) analyses were performed using the LEfSe tool (Segata et al., 2011). The relative abundances of bacteria were expressed as percentages.

Differences in endophytic bacterial abundance were analyzed by LDA EffectSize (LEfSe). LEfSe analysis uses the Kruskal–Wallis rank sum test to detect significantly different abundances and generates LDA scores to estimate the effect size (threshold: ≥3.5). Values are presented as the least-square means with standard errors of the mean. Differences were considered significant at *P*-values less than 0.05. Analysis of similarity (ANOSIM) was used to test the variation in microbial community composition. Principal component analysis (PCA) and non-metric multidimensional scaling (NMDS) were used to evaluate the differences among microbial communities. Differences were considered significant at *P*-values less than 0.001.

## Results

### Sample Collection, Sequencing, and Endophytic Profiles

A total of 30 root samples were collected from 10 sugarcane varieties, including *S. officinarum* (Badila and Black Cheribon), *S. robustum* (51NG208 and FJDY), *S. barberi* (HATUNI and Katha), and *S. spontaneum* (HN-83 and FJ88-1), along with two commercial sugarcane cultivars (ROC22 and YT93-159). Genomic DNA was successfully extracted, and the V3 and V4 regions of the 16S rRNA gene were amplified by polymerase chain reaction (PCR) using primers 341F and 806R. The raw data were submitted to the NCBI Sequence Read Archive (Accession no. SRP126795). The PCR products were sequenced to determine the endophytic community profiles. Sugarcane plastid sequences were removed from the data before microbial community analysis, leaving quality-filtered reads. The number of clean reads, mapped reads, and OTUs of each sample were summarized in **Supplementary Table S1**. Reads were clustered into a total of 7,198 OTUs, and each OTU comprised ≥2 reads, with a sequence threshold of 97% identity, corresponding to genus-level groupings. A total of 202 bacterial families were detected.

The *nifH* gene was amplified by PCR using primers Pol-F and Pol-R. However, only nine samples, from ROC22, Katah, Badila, Black Cheribon, FJ88-1, and HN-83, were successfully amplified and sequenced. The reads were merged into 1,734 OTUs for endophytic diazotrophs. A total of 43 bacterial families were detected.

**FIGURE 1:**
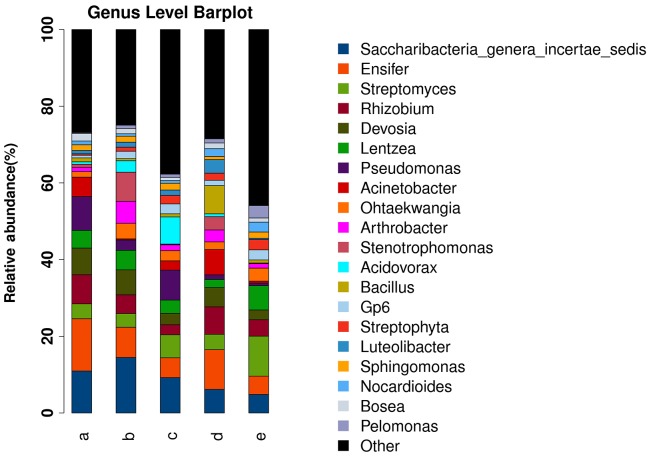
Histograms of the relative abundances at the genus level of the bacterial communities in the roots of different sugarcane species. The relative abundance (%) of the TOP 20 bacterial communities in the roots of four sugarcane species and commercial cultivars at the genus level are shown. (a) *S. officinarum*; (b) *S. barberi*; (c) *S. robustum*; (d) commercial cultivars; (e) *S. spontaneum*.

Relatively stringent quality control was used to avoid overestimation of the α-diversity of endophytic bacteria. Most erroneous sequences were singletons or doubletons. Although the total number of errors was low, they contributed to a large number of rare species and led to an overestimation of α-diversity (an estimation of the diversity in a sample or species richness). Diversity takes into account both taxon richness and evenness. The results demonstrated that these two parameters were highest in different sugarcane species (**Supplementary Figure S1**). The relative abundances of the microbial communities differed among the four sugarcane species analyzed. At the genus level, the most abundant microbes were *Saccharibacteria*, *Ensifer*, *Streptomyces*, *Devosia*, *Lentzea*, *Pseudomonas*, *Acinetobacter*, *Ohtaekwangia*, *Arthrobacter*, *Stenotrophomonas*, *Acidovorax*, *Bacillus*, *Gp6*, *Streptophyta*, *Luteolibacter*, *Sphingomonas*, *Nocardioides*, *Bosea*, and *Pelomonas*. In *S. officinarum* and *S. robustum*, *Saccharibacteria*, and *Ensifer* were the most abundant bacteria. *Ensifer* was the most abundant bacterial genus in the commercial sugarcane cultivars. In *S. robustum*, *Pseudomonas* was the most abundant genus, while *Streptomyces* and *Lentzea* were the most abundant genera in *S. spontaneum* (**Figure 1**). The top six phyla in the tested sugarcane samples were *Proteobacteria*, *Actinobacteria*, *Candidatus*, *Saccharibacteria*, *Bacteroidetes*, *Firmicutes*, and *Acidobacteria*.

### Effects of Host Genetics on α-Diversity

To compare the α-diversity of samples with different sequence counts, we refined the data (i.e., we randomly picked an equal number of sequences across samples) using Quantitative Insights into Microbial Ecology (QIIME). The rarefaction curves showed the richness of the observed OTUs (**Supplementary Figure S1A**) and indicated that the sequencing depth was sufficient to fully capture the diversity present. In addition to the OTU richness, total species richness was estimated using the Chao-1 estimator and the Shannon and Simpson indices, which yielded similar results. According to the box plot of the Shannon diversity indices, the diversity of the samples was as follows: *S. robustum* > *S. barberi* > *S. spontaneum* > *S. officinarum*> commercial cultivars; however, according to the Simpson diversity indices, the diversity of the samples was as follows: *S. barberi* > *S. robustum* > *S. officinarum* > commercial cultivars > *S. spontaneum* (**Supplementary Figure S1**). The four sugarcane species and cultivars were ranked according to the number of observed OTUs as follows: *S. robustum* > *S. spontaneum* > *S. barberi* > commercial cultivars > *S. officinarum*. *S. robustum* had the highest number of OTUs (*n* ≥ 3,700 OTUs, *P* < 8.00E-04) and *S. officinarum* had the lowest number of OTUs (*n* ≥ 2,100 OTUs, *P* < 8.00E-04).

### Effects of Host Genetics on β-Diversity

**FIGURE 2:**
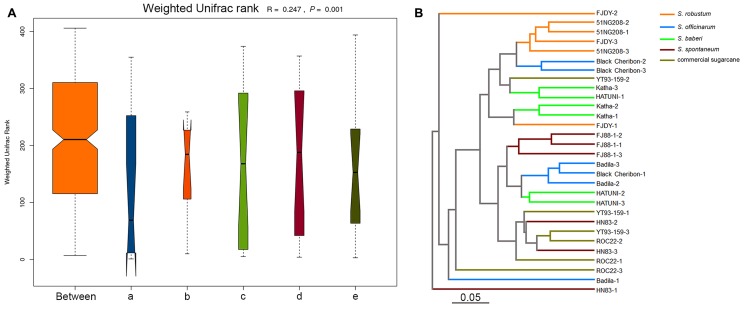
Beta-diversity analysis showing variation in the bacterial communities in different sugarcane species. The variation between different sugarcane species using ANOSIM and cluster analysis. **(A)** Analysis of similarity (ANOSIM) is a statistical test of the variation in microbial community composition, and the difference between the five sugarcane species was significantly greater than those in the group (*R* = 0.247, *P* = 0.001). (a) *S. officinarum*; (b) *S. barberi*; (c) *S. robustum*; (d) commercial cultivars; (e) *S. spontaneum*. **(B)** Unweighted pair group method with arithmetic mean (UPGMA) was used to form an evolutionary tree.

We used unweighted and weighted UniFrac distance measures to estimate β-diversity (the difference in species diversity between groups). NMDS plots based on Bray–Curtis distances were used to visualize the separation of the microbiota structures across different branches. Statistical testing of variation in microbial community composition was carried out using ANOSIM. Similarities between the bacterial communities of different sugarcane species were compared by ANOSIM and NMDS based on Bray–Curtis distance (Clarke, 1993). ANOSIM revealed significant differences in the structure of the root microbiota among different sugarcane species (**Figure 2A**; ANOSIM, *R* = 0.247, *P* = 0.001). Based on the results of the analysis, the eight ancestral sugarcane varieties and two commercial cultivars assayed had significantly different root microbial communities.

An evolutionary tree of all sugarcane species and cultivars was constructed using the unweighted pair-group method with arithmetic means (UPGMA; **Figure 2B**). No clear clades were observed according to the traditional classification of sugarcane germplasm as summarized by Wang et al. (2010) and Zhang et al. (2012). However, *S. robustum* and *S. officinarum* were classified into different groups (**Figure 2B**). One group was represented by *S. robustum*, and the other was represented by *S. spontaneum* (**Figure 2B**). Two replicates of Black Cheribon (*S. officinarum*) clustered into the *S. robustum* group, while the other *S. officinarum* species clustered into the *S. spontaneum* group. Three *S. barberi* samples clustered into the *S. robustum* group. Most of the commercial cultivars clustered into the *S. spontaneum* group, while only one sample clustered into the *S. robustum* group.

Based on the Venn diagram of the OTUs from the four sugarcane species, *S. robustum* and *S. officinarum* had the most and least OTUs, respectively, while the OTUs of the commercial cultivars ROC22 and YT93-159 were similar to those of *S. officinarum* (**Supplementary Figure S2**). NMDS is a data analysis method that simplifies the research objects (samples or variables) of multidimensional space into lower dimensional space for location, analysis, and classification, while retaining the original relationship between objects. For the four sugarcane species, *S. barberi* and *S. robustum* were different from the sugarcane cultivars and *S. spontaneum* in MDS1 (**Supplementary Figure S2**, NMDS with ADONIS test: *R*^2^ = 0.31826, *P* = 0.001). *S. spontaneum* and commercial sugarcane were different from the other three species (**Supplementary Figure S2B**).

**FIGURE 3:**
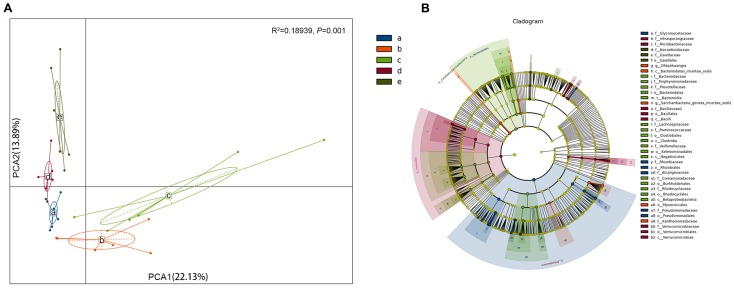
Analysis of the bacterial species in the roots of different sugar cane species shows significant differences in the endophytic bacteria communities. **(A)** Principal component analysis (PCA) shows the grouping patterns of the four sugarcane species based on weighted UniFrac distance. Each colored dot represents a sample. Adonis test: *R*^2^ = 0.18939, *P* = 0.001. **(B)** Linear discriminant analysis (LDA) and effect size (LEfSe) analysis were used to determine the significant discriminative taxa between the four sugarcane species (LDA score threshold: ≥3.5). Different colored regions represent different species. The circles from inside to out represent the classification levels from the phylum to genus (or species). Each small filled circle represents a classification at this level, and size is proportional to relative abundance. (a) *S. officinarum*; (b) *S. barberi*; (c) *S. robustum*; (d) commercial cultivars; (e) *S. spontaneum*.

The rank sum test was used to analyze the significance of differences among groups. Using the Kruskal–Wallis test, 242 species (at all levels) exhibited obvious differences among groups (*P* < 0.05). Based on the PCA analysis of the different bacterial species observed in the four different sugarcane species, *S. barberi* and *S. robustum* were different from *S. officinarum*, the commercial cultivars, and *S. spontaneum* (in the first principal component there is a 22.13% contribution to the difference; **Figure 3A**; ADONIS test: *R*^2^ = 0.18939, *P* = 0.001). In the five groups, at all levels and the genus level different TOP20 species were observed (**Supplementary Figures S3A,B**).

### Bacterial Groups with Significant Differences

In addition to the α- and β-diversities, another primary purpose of comparing the microbial communities is to identify specialized bacterial groups within each type of sample. LEfSe was used for statistical analysis of the metagenomic data from two or more microbial communities (Segata et al., 2011). This method is used to analyze data in which the number of species is much higher than the number of samples and to provide biological class explanations to establish statistical significance, biological consistency, and effect-size estimation of predicted biomarkers (Segata et al., 2011). This tool can be used to analyze bacterial community data at any taxonomic level. However, due to the extensive analysis of OTUs in the current study, the calculation is too dense; therefore, we only performed a statistical analysis from the domain to genus levels. The top 20 OTUs (at both the total and genus levels) in the four sugarcane species were very different (**Supplementary Figure S3**).

A total of 237 distinct bacterial groups were identified using the default logarithmic (LDA) value of 2 (**Supplementary Figure S4**). Cladograms show taxa with LDA values higher than 3.5 for clarity (**Figure 3B**), and the LDA value for each lineage is listed in **Supplementary Figure S4**. Thirty-nine groups of bacteria were enriched in the four sugarcane species, and 275 biomarkers were identified. The *S. officinarum* microbiome was characterized by the presence of *Proteobacteria*, *Rhizobiales*, *Rhizobiaceae*, *Pseudomonadales*, *Ensifer*, *Pseudomonadaceae*, *Pseudomonas*, *Rhizobium*, *Devosia*, *Alcaligenaceae*, and *Achromobacter* [LAD(log_10_) > 4.0]; *S. barberi* was characterized by the presence of *Saccharibacteria*, *Candidatus*, *Xanthomonadaceae*, and *Stenotrophomonas* [LAD(log_10_) > 4.0]; *S. robustum* was characterized by the presence of *Betaproteobacteria*, *Burkholderiales*, *Bacteroidetes*, *Bacteroidia*, *Bacteroidales*, *Acidovorax*, *Comamonadaceae*, *Clostridia*, and *Clostridiales* [LAD(log_10_) > 4.0]; and the commercial sugarcane cultivars were characterized by the presence of *Firmicutes*, *Bacilli*, *Bacillales*, *Bacillaceae*, *Bacillus*, *Verrucomicrobiae*, *Verrucomicrobiaceae*, *Verrucomicrobiales* [LAD(log_10_) > 4.0]. Interestingly, no bacteria had a LAD (log_10_) greater than 4.0 for *S. spontaneum*. The *Rhizobiales* and *Proteobacteria* were particularly enriched in the *Rhizobium* genus (**Supplementary Figure S4**).

**FIGURE 4:**
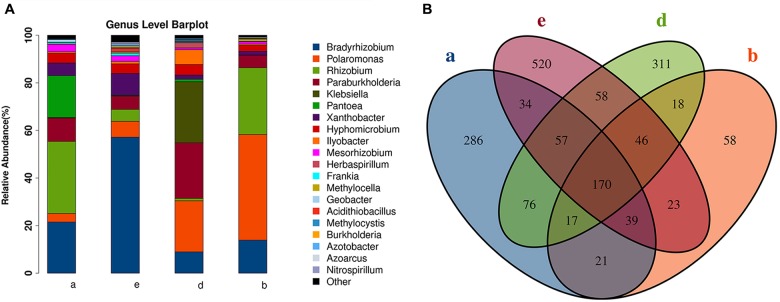
Diagram illustrating overlap among the root-associated core bacterial communities at the family level shared among four sugarcane species (this study), sugarcane cultivars grown under varying nitrogen fertilizer rates, and *A. thaliana*.

Despite the strong influence of the sugarcane germplasm at species level, a core microbiome was identified in the tested sugarcane roots. The core microbiome contains the following 13 bacterial families: *Intrasporangiaceae*, *Burkholderiales incertae sedis*, *Sphingomonadaceae*, *Micromonosporaceae*, *Rhodospirillaceae*, *Erythrobacteraceae*, *Phyllobacteriaceae*, *Chitin -ophagaceae*, *Nocardioidaceae*, *Microbacteriaceae*, *Comamona -daceae*, *Oxalobacteraceae*, and *Enterobacteriaceae*. Eight bacterial families of the core microbiome overlapped with the core microbiome identified in sugarcane by Yeoh et al. (2016) and 10 overlapped with the core microbiome identified in *Arabidopsis* (Bulgarelli et al., 2012; Lundberg et al., 2012; Natacha et al., 2013; Schlaeppi et al., 2014). Seven bacterial families were present in all three core microbiomes (**Figure 4**).

### Influence of Host Genetics on Root Diazotrophs

**FIGURE 5:**
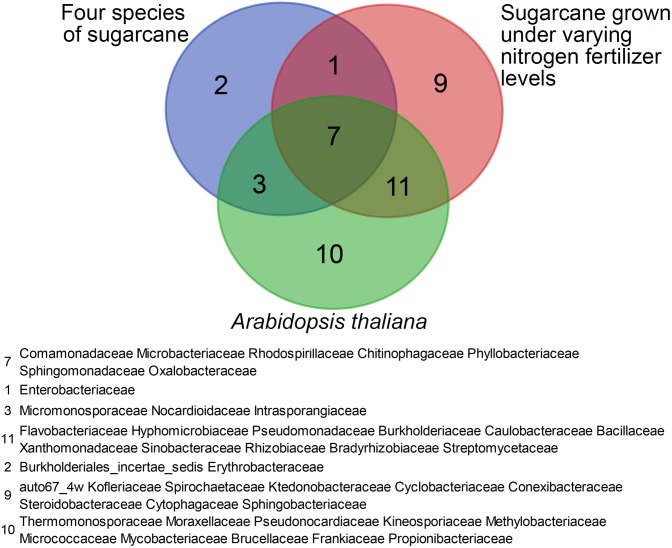
Endophytic diazotrophs detected in sugarcane roots by *nifH* gene analysis. **(A)** The relative abundances of the TOP 20 diazotrophs in four species of sugarcane at the genus level. **(B)** A Venn diagram of operational taxonomic units (OTUs) among three sugarcane species. (a) *S. officinarum*; (b) *S. barberi*; (d) commercial cultivars; (e) *S. spontaneum*.

We used the *nifH* gene to identify diazotrophs in the sugarcane roots. However, the *nifH* gene was successfully amplified in only nine samples from three species (*S. spontaneum*, *S. barberi*, and *S. officinarum*) and cultivar ROC22. A total of 43 families of endophytic diazotrophs were detected. Among the top 20 most abundant genera (**Figure 5A**), 10 were reported to be involved in nitrogen fixation in sugarcane. Among the 1,734 OTUs, 170 were found to be present in all sugarcane samples containing a successfully sequenced *nifH* gene (**Figure 5B**); *S. spontaneum* had the most OTUs, and *S. barberi* had the least. Based on the identified OTUs by *nifH* gene sequencing, *S. spontaneum* and *S. officinarum* were roughly grouped into different clades, and the commercial sugarcane cultivar ROC22 formed another separate clade (**Supplementary Figures S5A,B**).

## Discussion

The microbiomes of plant roots are integral to host plant function (Weyens et al., 2009; Panke-Buisse et al., 2015). Microorganisms associated with root systems have been studied using both culture-dependent and culture-independent 16S rRNA-based methods, such as denaturing gradient gel electrophoresis (DGGE), terminal restriction fragment length polymorphism (TRFLP), and 16S rRNA clone libraries, which provide a better overview of the *in situ* populations than culture-based methods (Amann et al., 1995). Using next-generation sequencing platforms, culture-independent molecular methods provide high-resolution microbial community profiles (Lebeis et al., 2012). The first example of high-resolution analysis of the root microbiome was performed in the model plant *A. thaliana* (Bulgarelli et al., 2012; Lundberg et al., 2012; Bodenhausen et al., 2013; Schlaeppi et al., 2014).

In Brazil, low nitrogen fertilizers are applied during sugarcane production (Li and Yang, 2015). In some sugarcane cultivars, diazotrophs could provide 70% of the required nitrogen. This has led to intensive studies to identify sugarcane diazotrophs. Many diazotrophs have been identified using culture-dependent and -independent methods (Yeoh et al., 2016). We speculate that sugarcane germplasm is the primary determinant of the overall bacterial community composition, as nitrogen fertilizer rates, location, and developmental stage had no obvious influence on the microbiome of sugarcane cultivars (Yeoh et al., 2016). This idea is supported by studies on *A. thaliana* (Bulgarelli et al., 2012; Lundberg et al., 2012) and *Zea mays* (Peiffer et al., 2013), and partially supported by studies on potato (Manter et al., 2010).

The present survey of the root-associated (endophyte) communities of field-grown sugarcane is, to the best of our knowledge, the first to investigate the effects of host genotype on root bacterial community composition through high-resolution community profiling of original species and commercial cultivars. In this study, we characterized the endophytic microbial community composition of four *Saccharum* species (eight total genotypes) and cultivars (two genotypes) planted in the NNSGR (Kaiyuan, China) during the grand growth phase. We also profiled the endophytic diazotroph communities in the roots of four sugarcane species based on *nifH* gene sequences (Poly et al., 2001). Our experimental design allowed us to assess the influence of sugarcane host genotype on the endophyte and diazotroph microbial communities. Based on the α- and β-diversities, sugarcane genotype significantly influenced the bacterial community structure (**Figure 2A** and **Supplementary Figure S2B**). Modern sugarcane cultivars were bred about a century ago by interspecific crosses between *S. officinarum* and *S. spontaneum* and a backcross to *S. officinarum*. Conventionally, the genus *Saccharum* is composed of six species, *S. spontaneum*, *S. robustum*, *S. officinarum*, *S. barberi*, *S. sinense*, and *S. edule*; *S. spontaneum* and *S. robustum* are wild species (D’Hont et al., 1996, 2002; Ha et al., 1999), *S. officinarum* is likely derived from *S. robustum* (Lu et al., 1994; Schenck et al., 2004; D’Hont et al., 2011), and *S. barberi*, *S. sinense*, and *S. edule* are interspecific hybrids (D’Hont et al., 2002). However, we are lack of information on the extent of genetic diversity among the plants used in the present study. Although UPGMA demonstrated that a dendrogram based on the bacterial communities of the eight sugarcane varieties and two commercial cultivars differed from a previous one based on RFLP markers (Lu et al., 1994), the bacterial communities of *S. robustum* and *S. spontaneum* were clustered into two different groups (**Figure 2B**), which coincides with the classification of sugarcane germplasm and supported the view that sugarcane germplasm influences the bacterial community.

Yeoh et al. (2016) identified a core set of microbial taxa shared by sugarcane and *A. thaliana*. In an investigation of the bacterial communities in the roots of field-grown sugarcane subjected to industry-standard or reduced nitrogen fertilizer application, a core group of sugarcane root-enriched bacterial families was also found, despite the large differences in the soil microbial communities between test sites (Yeoh et al., 2016). In this study, enrichment of a core set of microbial taxa in sugarcane root communities (13/202 bacteria families) was observed, again despite significant differences in the microbial community composition of the roots of the eight sugarcane varieties and two commercial cultivars (**Figure 4**). Seven bacterial families of the core community identified in this study overlapped with the core bacterial communities previously identified in *A. thaliana* and the sugarcane cultivars grow under regular or reduced nitrogen conditions (**Figure 4**). These results support the speculation that certain bacterial families have been long associated with plants (Yeoh et al., 2016).

We observed a few taxa that were consistently enriched in the sugarcane roots, including *Saccharibacteria*, *Ensifer*, *Streptomyces*, *Rhizobium*, *Devosia*, *Lentzea*, *Pseudomonas*, *Acinetobacter*, *Ohtaekwangia*, *Arthrobacter*, and *Streptophyta*. In a previous study, *Streptomyces*, *Rhizobium*, and *Acinetobacter* were shown to be enriched in the sugarcane endophyte community (Velázquez et al., 2008; Rouws et al., 2014; Kruasuwan et al., 2017). Recent studies on *Streptomyces* have produced a draft genome sequence, and have shown it to be a plant growth-promoting endophyte (Kruasuwan et al., 2017). Although in our study, most bacterial taxa of lower rank were not sequenced at sufficiently high coverage to enable powerful comparisons, we did observe enrichment of the genus *Pseudomonas*, members of which are well-known plant growth-promoting endophytic bacteria. For example, *Pseudomonas* species have been used to alleviate heavy metal toxicity caused by the application of plant growth-promoting rhizobacteria and the negative effects of saline sodic field growth on wheat (Hassan et al., 2017). Taken together, these results underscore the fact that *Streptomyces* are generally adapted as endophytes across diverse plant species. This finding is not surprising, as *Streptomyces* are well-known to have complex secondary metabolism (McCormick and Flärdh, 2012; Huang et al., 2015), producing over two-thirds of the clinically useful antibiotics of natural origin (e.g., neomycin, cypemycin, grisemycin, bottromycin, and chloramphenicol) (Bibb, 2013).

Although we attempted to detect the presence of diazotrophs in sugarcane roots using the *nifH* gene, diazotrophs were not detected in all samples. The nitrogen fertilizers rate used in this study was 350 kg/ha, a conventional practice for sugarcane production in China. However, it is very high compared with the nitrogen fertilizers rate used in Brazil and Australia. Under such high rate of nitrogen fertilizers, the low level of nitrogen fixers possibly don’t have any advantage over non-fixers. Based on the results obtained from three sugarcane species, we observed that a significant fraction of the variation in microbial diversity could be attributed to host genetics. *Bradyrhizobium* was the top genus in the three sugarcane species, and bacteria in this genus showed nitrogenase activity in an acetylene reduction assay, suggesting that they do not require a nodule environment for nitrogen fixation (Rouws et al., 2014).

In summary, this study provides evidence for genetic variation in the bacterial and diazotroph communities among different sugarcane species. However, a number of questions remain: what specific sugarcane alleles are responsible for this microbial variation, what phenotypic differences do they encode; and finally, what is the effect of these different phenotypes on microbial diversity. Studies using a larger, more diverse sugarcane population that are focused on sequencing the existing endophytic microbial diversity are necessary to better describe the symbiotic relationships under natural environmental conditions. Future research should also focus on the functional microbial groups rather than the taxonomic relationships of the microbial communities.

## Author Contributions

MD and JX designed the research and wrote the paper. MD and ZY prepared the materials. MD, GC, LP, and QX analyzed the data.

## Conflict of Interest Statement

The authors declare that the research was conducted in the absence of any commercial or financial relationships that could be construed as a potential conflict of interest.

## Supplementary Material

FIGURE S1 **(A)** The rarefaction curves of 30 sugarcane samples. **(B)** Boxplots for α-diversity metrics of Shannon diversity index. **(C)** Boxplots for α-diversity metrics of Simpson diversity index. (a) *S. officinarum*; (b) *S. barberi*; (c) *S. robustum*; (d) commercial cultivars; (e) *S. spontaneum*.

FIGURE S2 Endophytic bacterial diversity among four sugarcane species. **(A)** Venn diagram showing the OTUs of the endophytic bacterial communities in the roots of four sugarcane species. **(B)** NMDS analysis illustrating the grouping patterns of the four sugarcane species based on weighted UniFrac distances. Each colored dot represents a sample. Adonis test: *R*^2^ = 0.31826, *P* = 0.001. (a) *S. officinarum*; (b) *S. barberi*; (c) *S. robustum*; (d) commercial cultivars; (e) *S. spontaneum*.

FIGURE S3 Boxplot results of the top 20 OTUs in the sugarcane species (**A** is at the total level, **B** is at the genus level).

FIGURE S4 Bar diagram of the linear discriminant analysis (LDA) distribution (LDA score threshold: ≥2).

FIGURE S5 The distribution of diazotrophs in the roots of three sugarcane species. **(A)** Cluster diagram of the abundances of the TOP20 species. **(B)** Heat map showing the abundances of each diazotroph in different samples. (a) *S. officinarum*; (b) *S. barberi*; (c) *S. robustum*; (d) commercial cultivars; (e) *S. spontaneum*.

TABLE S1 The summary of each sample OTUs.
