# Supplementary figures and images for: Diversity of the Bacterial Microbiome in the Roots of Four Saccharum Species: S. spontaneum, S. robustum, S. barberi, and S. officinarum
Source: Front Microbiol. 2018 Feb 21;9:267. doi: 10.3389/fmicb.2018.00267 (PMC5826347; doi:10.3389/fmicb.2018.00267)

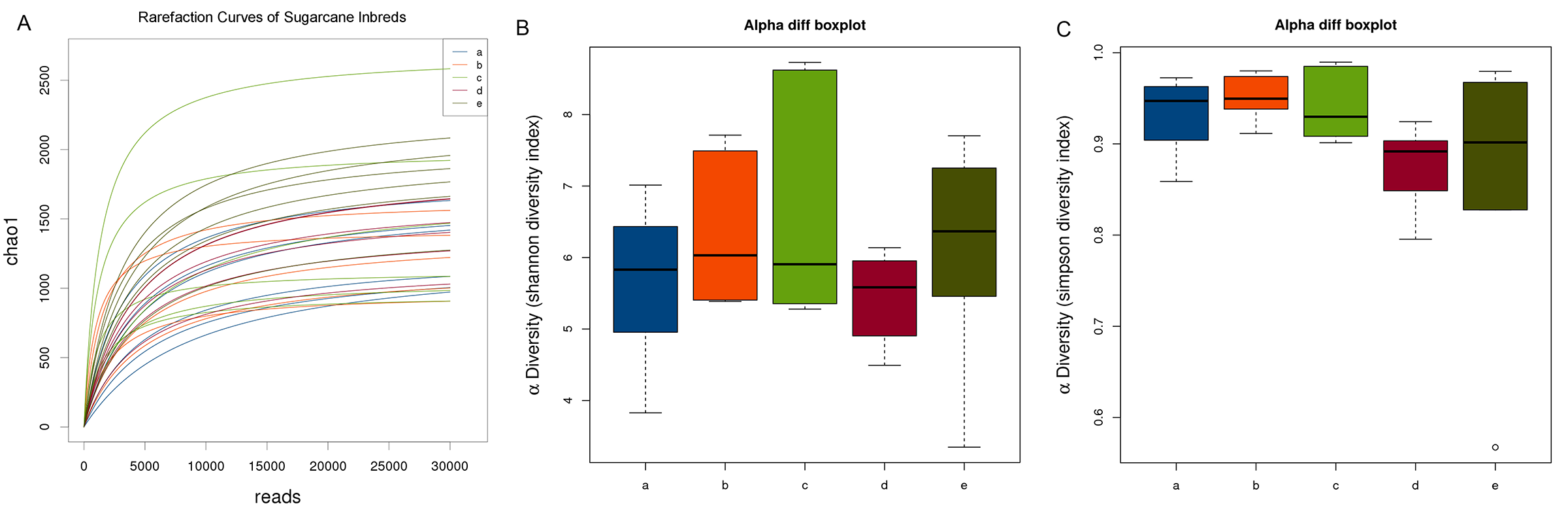

Supplement: FIGURE S1 — (A) The rarefaction curves of 30 sugarcane samples. (B) Boxplots for α-diversity metrics of Shannon diversity index. (C) Boxplots for α-diversity metrics of Simpson diversity index. (a) S. officinarum; (b) S. barberi; (c) S. robustum; (d) commercial cultivars; (e) S. spontaneum. [file Image_1.TIF]

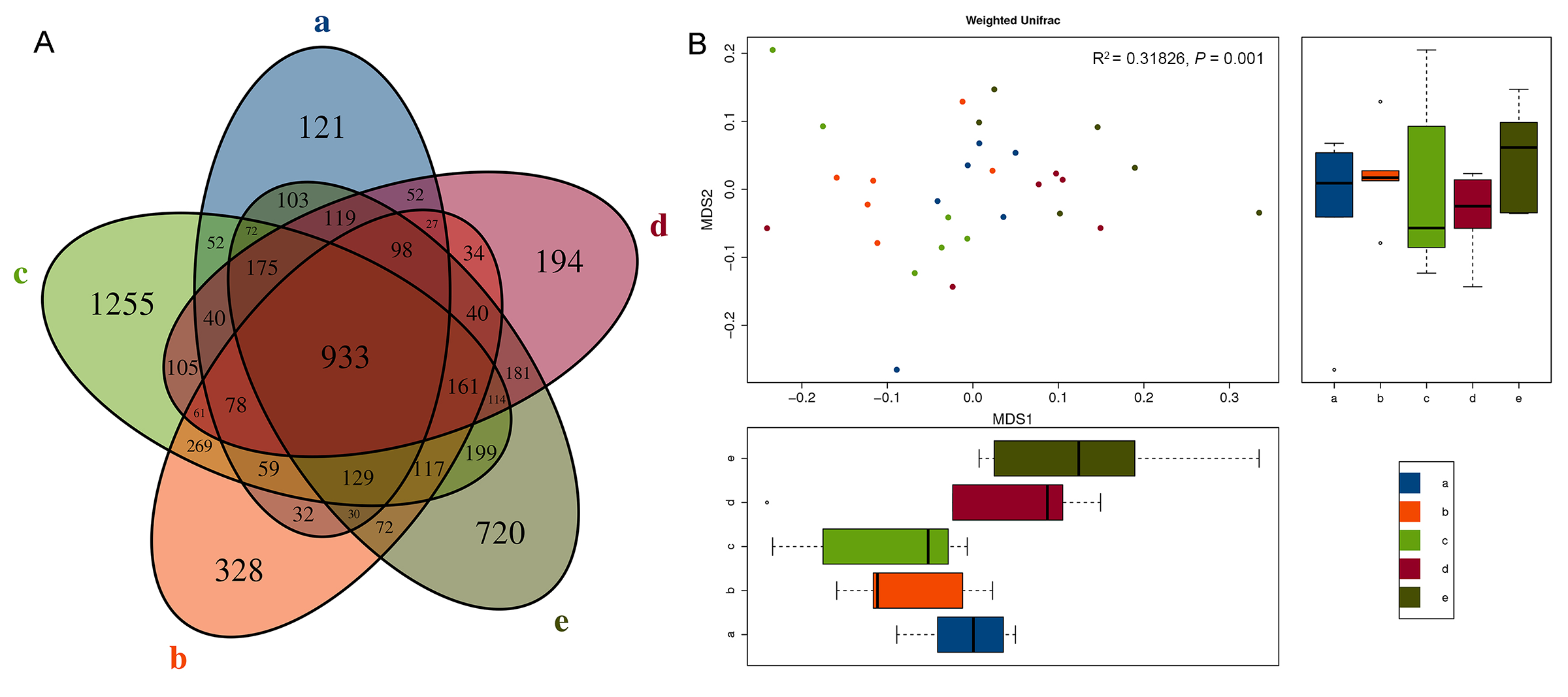

Supplement: FIGURE S2 — Endophytic bacterial diversity among four sugarcane species. (A) Venn diagram showing the OTUs of the endophytic bacterial communities in the roots of four sugarcane species. (B) NMDS analysis illustrating the grouping patterns of the four sugarcane species based on weighted UniFrac distances. Each colored dot represents a sample. Adonis test: R2 = 0.31826, P = 0.001. (a) S. officinarum; (b) S. barberi; (c) S. robustum; (d) commercial cultivars; (e) S. spontaneum. [file Image_2.TIF]

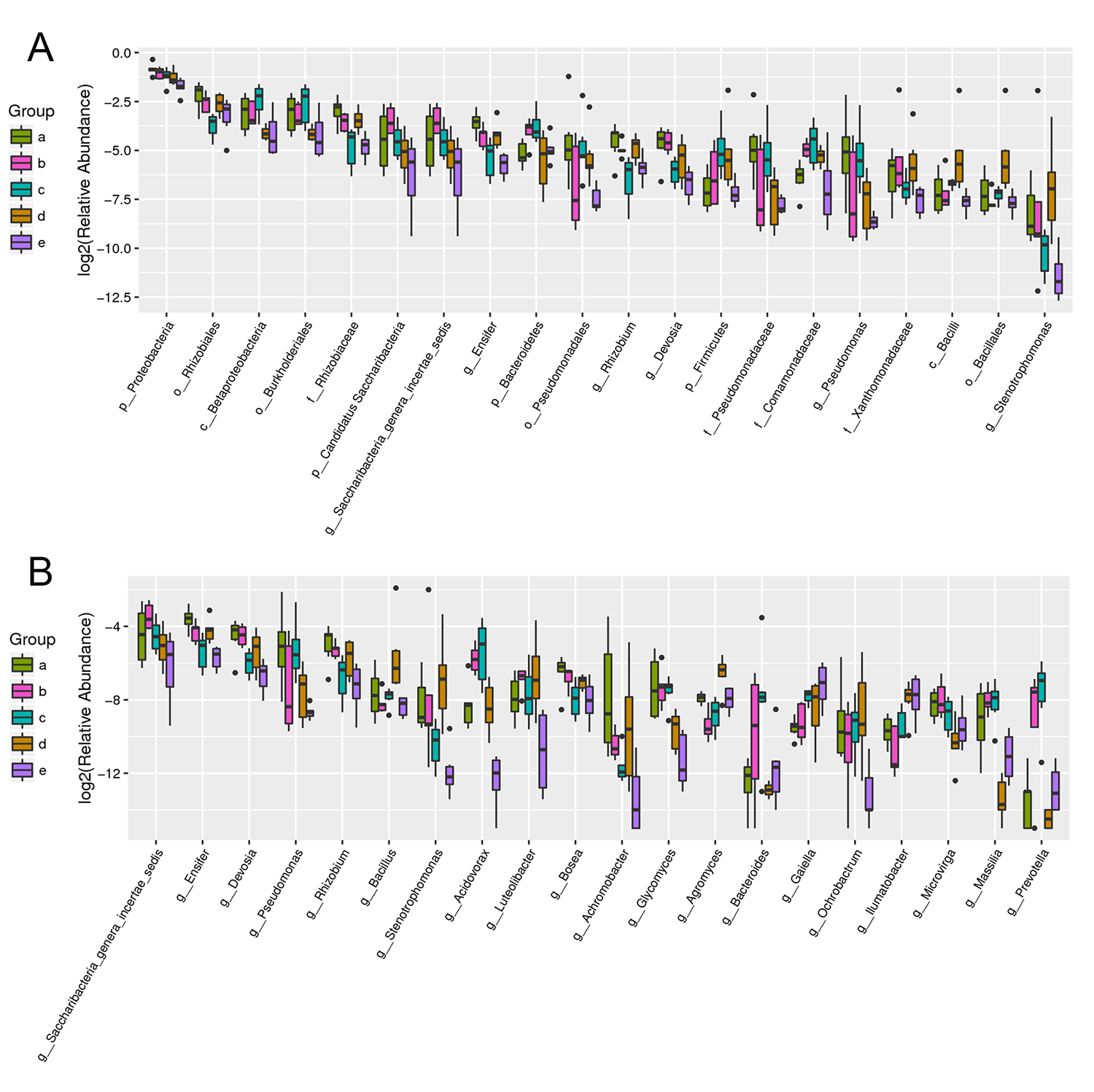

Supplement: FIGURE S3 — Boxplot results of the top 20 OTUs in the sugarcane species (A is at the total level, B is at the genus level). [file Image_3.TIF]

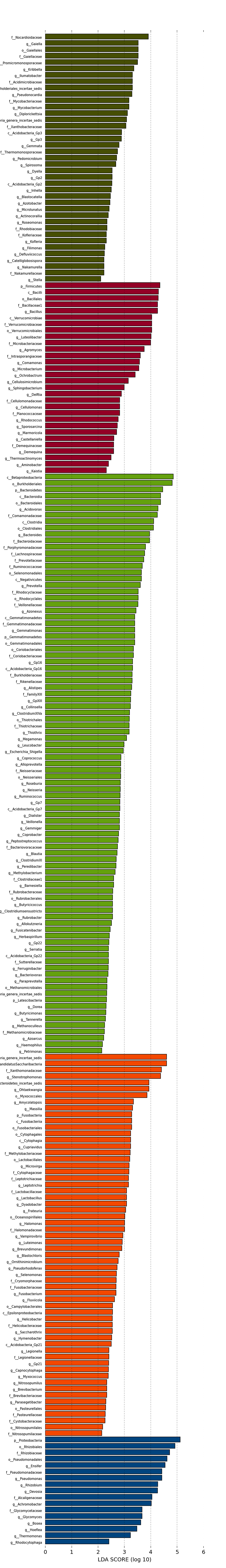

Supplement: FIGURE S4 — Bar diagram of the linear discriminant analysis (LDA) distribution (LDA score threshold: ≥2). [file Image_4.TIF]

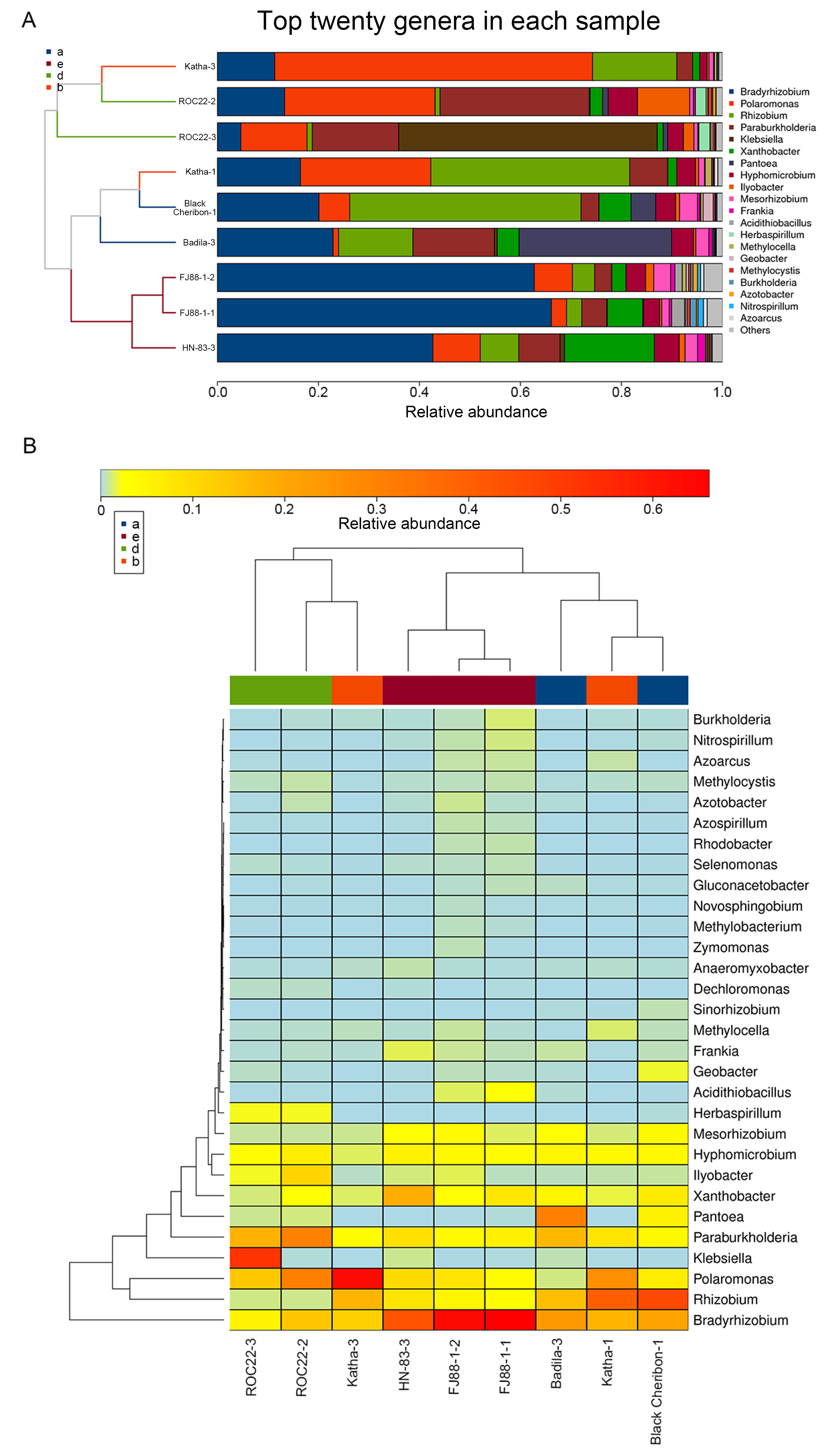

Supplement: FIGURE S5 — The distribution of diazotrophs in the roots of three sugarcane species. (A) Cluster diagram of the abundances of the TOP20 species. (B) Heat map showing the abundances of each diazotroph in different samples. (a) S. officinarum; (b) S. barberi; (c) S. robustum; (d) commercial cultivars; (e) S. spontaneum. [file Image_5.TIF]
