# Supplementary material for: Diversity of the Bacterial Microbiome in the Roots of Four Saccharum Species: S. spontaneum, S. robustum, S. barberi, and S. officinarum
Source: Front Microbiol. 2018 Feb 21;9:267. doi: 10.3389/fmicb.2018.00267 (PMC5826347; doi:10.3389/fmicb.2018.00267)
Supplement: TABLE S1 — The summary of each sample OTUs. [file Table_1.docx]

| **Table S1. The summary of each sample OTUs** | | | | |
| --- | --- | --- | --- | --- |
| **Samples** | **Clean reads** | **Mapped reads** | **Mapped ratio (%)** | **OTUs** |
| Badila-1 | 111577 | 90031 | 80.68956864 | 908 |
| Badila-2 | 114730 | 84718 | 73.84119236 | 1456 |
| Badila-3 | 120711 | 93680 | 77.6068461 | 1788 |
| FJ88-1-1 | 112383 | 79109 | 70.39231912 | 2004 |
| FJ88-1-2 | 126873 | 100514 | 79.224106 | 2265 |
| FJ88-1-3 | 123546 | 82172 | 66.51125896 | 2040 |
| FJDY-1 | 126680 | 106307 | 83.9177455 | 2792 |
| FJDY-2 | 87886 | 75707 | 86.14227522 | 2029 |
| FJDY-3 | 85080 | 80747 | 94.90714622 | 1212 |
| HATUNI-1 | 114065 | 106799 | 93.62994784 | 1032 |
| HATUNI-2 | 127964 | 106289 | 83.06164234 | 1373 |
| HATUNI-3 | 120024 | 102586 | 85.47123909 | 1152 |
| Black Cheribon-1 | 119315 | 97626 | 81.82206764 | 1540 |
| Black Cheribon-2 | 127087 | 101505 | 79.87048243 | 1094 |
| Black Cheribon-3 | 125136 | 96626 | 77.21678813 | 1179 |
| HN83-1 | 126247 | 113189 | 89.65678392 | 1511 |
| HN83-2 | 120057 | 98839 | 82.32672814 | 1955 |
| HN83-3 | 115660 | 98213 | 84.91526889 | 1789 |
| Katha-1 | 39970 | 36436 | 91.15836878 | 1488 |
| Katha-2 | 35995 | 32691 | 90.82094735 | 1353 |
| Katha-3 | 37979 | 34558 | 90.58065982 | 1417 |
| 51NG208-1 | 52756 | 46669 | 88.46197589 | 1164 |
| 51NG208-2 | 46010 | 44400 | 96.5007607 | 944 |
| 51NG208-3 | 38363 | 36433 | 94.96911086 | 860 |
| ROC22-1 | 129891 | 103223 | 79.46893934 | 1587 |
| ROC22-2 | 116597 | 76100 | 65.26754548 | 1637 |
| ROC22-3 | 118455 | 87181 | 73.5984129 | 1153 |
| YT93-159-1 | 127764 | 106858 | 83.63701825 | 1725 |
| YT93-159-2 | 119381 | 104444 | 87.48795872 | 1405 |
| YT93-159-3 | 116106 | 89287 | 76.90127987 | 1748 |
